# Preparation of siRNA–PLGA/Fabʹ–PLGA mixed micellar system with target cell-specific recognition

**DOI:** 10.1038/s41598-021-96245-3

**Published:** 2021-08-18

**Authors:** Mai Hazekawa, Takuya Nishinakagawa, Takeshi Mori, Miyako Yoshida, Takahiro Uchida, Daisuke Ishibashi

**Affiliations:** 1 Department of Immunological and Molecular Pharmacology, Faculty of Pharmaceutical Sciences, Fukuoka University, 8-19-1 Nanakuma, Jonan-ku, Fukuoka, 814-0180 Japan; 2 Department of Clinical Pharmaceutics, Faculty of Pharmaceutical Sciences, Mukogawa Women’s University, 11-68 Koshien, 9-Bancho, Nishinomiya, Hyogo 663-8179 Japan

**Keywords:** Drug delivery, Pharmaceutics

## Abstract

Small interfering RNAs (siRNAs) are susceptible to nucleases and degrade quickly in vivo. Moreover, siRNAs demonstrate poor cellular uptake and cannot cross the cell membrane because of its polyanionic characteristics. To overcome these challenges, an intelligent gene delivery system that protects siRNAs from nucleases and facilitates siRNA cellular uptake is required. We previously reported the potential of siRNA-poly(d,l-lactic-co-glycolic acid; PLGA) micelles as an effective siRNA delivery tool in a murine peritoneal dissemination model by local injection. However, there was no effective formulation for siRNA delivery to target cells via intravenous injection. This study aimed to prepare siRNA–PLGA/Fabʹ–PLGA mixed micelles for siRNA delivery to target floating cells and evaluate its formulation in vitro. As the target siRNA protein in CEMx174, CyclinB1 levels were significantly reduced when siRNA–PLGA/Fabʹ–PLGA mixed micelles were added to cells compared with siRNA–PLGA micelles. siRNA–PLGA/Fabʹ–PLGA mixed micelles have high cell permeability and high target cell accumulation by endocytosis because flow cytometry detected labeling micelles in target cells. This study supports siRNA–PLGA/Fabʹ–PLGA mixed micelles as an effective siRNA delivery tool. This formulation can be administered systemically in dosage form against target cells, including cancer metastasis or blood cancer.

## Introduction

A small interfering RNA (siRNA) can recognize and degrade its complementary target mRNA in a sequence-specific manner at the post-transcriptional level^1, 2^. Use of RNA interference (RNAi)^3^ to suppress target gene expression has widespread therapeutic potential. siRNA can target genes that are specific for tumor cells, thus leaving healthy, non-tumor tissue unaffected. However, developing safe and efficient carriers for siRNA delivery remains a significant challenge in animal experiments and clinical trials.

In our previous study, we developed a drug-delivery system using poly(d,l-lactic-co-glycolic acid (PLGA), which maintained the stability of the encapsulated drug and enabled the regulation of drug release^4, 5^. Additionally, our recent research demonstrated that polyethylenimine (LPEI)-coated siRNA–PLGA micelles were useful and safe as a siRNA delivery system by intraperitoneal injection in a mouse peritoneal dissemination model of ovarian cancer^6^; however, its specific accumulation for targeting cells by intravenous injection remains unclear.

Generally, small nanoparticles (20–100 nm), including micelles, actively accumulate in tumor tissues through the enhanced permeability and retention (EPR) effect, which is characterized by leaky vasculature and impaired lymphatic drainage surrounding a tumor^7, 8^. However, expecting sufficient accumulation in cancer tissue by simply using nanoparticles is unreasonable, and there is no universal formulation design for targeting carriers because the EPR effect substantially varies depending on the patient and cancer type^9–11^. Attaching targeting ligands to nanoparticles or modifying the surface of nanoparticles provides further advantages for drug delivery, such as high-performance targeting, increased cellular uptake, and improved therapeutic efficacy^12–15^.

Numerous targeting molecules are available for attachment to nanoparticles, including small molecules, peptides, monoclonal antibodies, engineered proteins, and nucleic acid aptamers. Choosing the correct targeting ligand and conjugation chemistry is essential for a universal formulation design and may impact the therapeutic outcome^16, 17^. Antibody-based cell-targeting molecules, such as mAb, F(abʹ)2, antibody-binding fragment Fab, Fabʹ, and scFv, can potentially be used as the binding component in targeted nanoparticles because antibodies can be generated against a wide variety of targets with controlled affinity and specificity. Furthermore, these molecules can be produced in high yields using a cell-based expression system. There is also an advantage to reducing the particle size; because antibody fragments are smaller compared with the original size of antibodies, delivery through membranes by smaller particles is more effective. Using antibody fragments can also reduce non-specific binding by eliminating Fc interactions because many cells have receptors that can bind to the Fc region.

**Figure 1:**
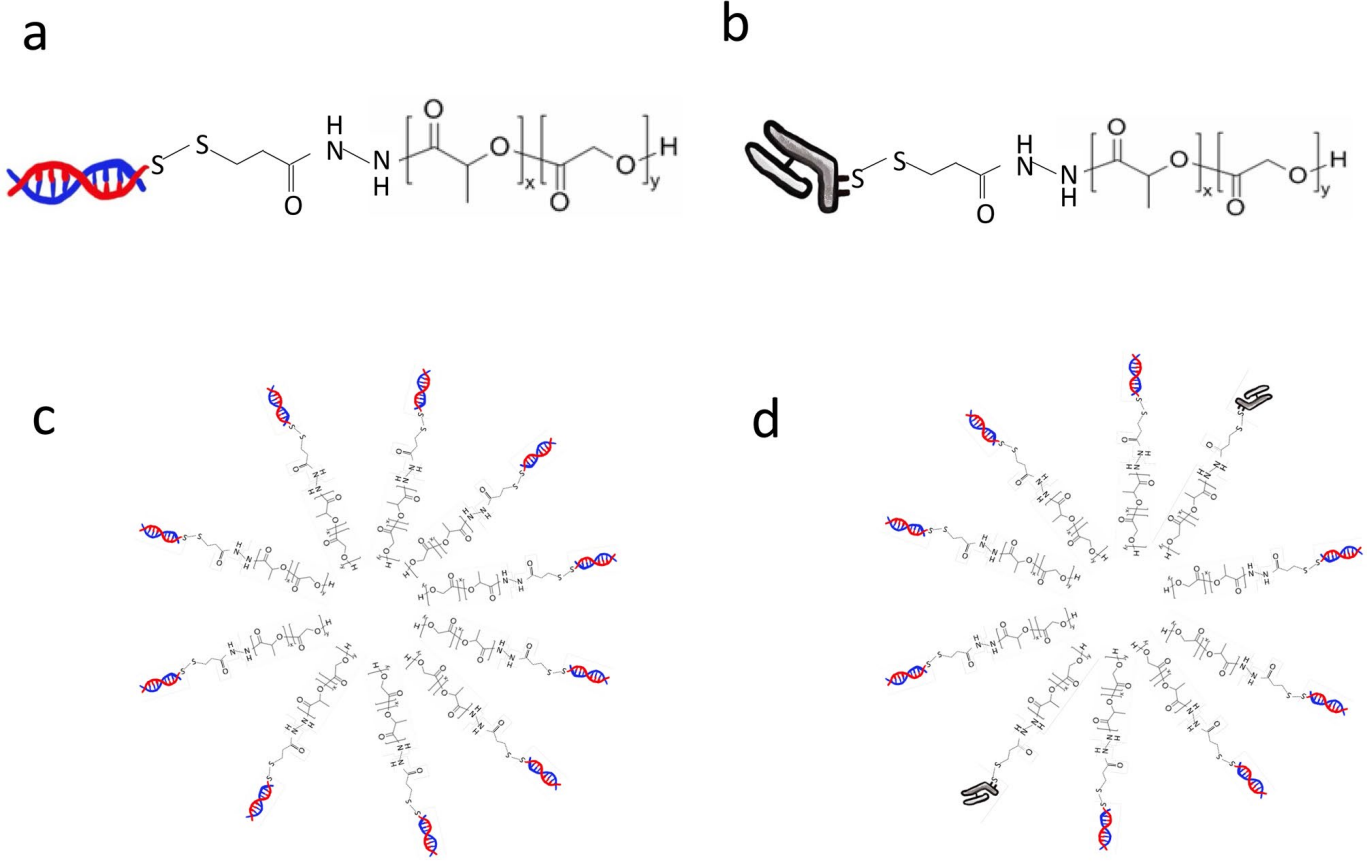
Structure of the siRNA–PLGA (**a**) and Fabʹ–PLGA hybrids (**b**). The siRNA–PLGA hybrid (**a**) self-associates above the critical micelle concentration to form (**c**) in an aqueous solution. Form (**d**) was formed by self-assembly when (**a**) was mixed with (**b**) in an aqueous solution.

In this study, we followed our previous methods to prepare mixed micelles comprising the siRNA–PLGA and Fabʹ–PLGA hybrids (Fig. 1). The CEMx174 cell line was used as a model human floating cell line because the purpose of this study was to develop an antibody-based drug carrier that demonstrates accumulation in systemic circulation. Thus, the formulation was evaluated in vitro for targeting and intracellular uptake efficiency. This is the first paper that proposes this novel mixed-micelles type of formulation using antibody fragments for drug targeting in siRNA delivery.

## Results

### Characterization of siRNA–PLGA conjugate and Fab′–PLGA conjugate

**Figure 2:**
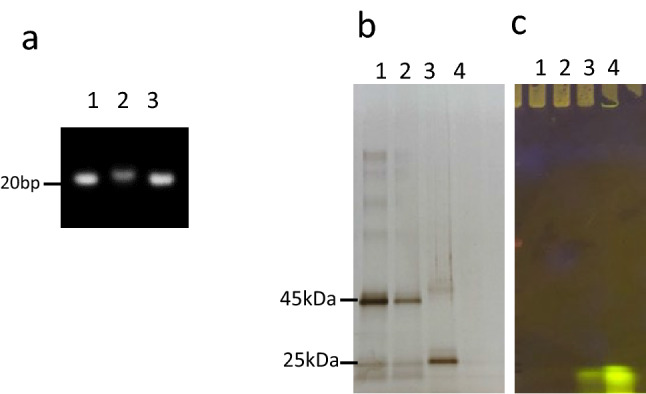
Characterization of siRNA–PLGA and Fab′–PLGA conjugates via covalent bond using agarose gel electrophoresis and SDS-PAGE. (**a**) 4% agarose gel electrophoresis to detect siRNA in siRNA–PLGA conjugate by ethidium bromide staining. Lane1: siRNA, Lane2: siRNA–PLGA, Lane3: siRNA–PLGA treated with DTT. (**b**) SDS-PAGE to detect Fab′ in Fab′–PLGA by Ag staining. Lane1: Fab′, Lane2: Fab′–PLGA(FITC), Lane3: Fab′–PLGA(FITC) treated with DTT, Lane4: PLGA(FITC). (**c**) SDS-PAGE to detect FITC derived FITC labeling PLGA using transilluminator. Lane1: Fab′, Lane2: Fab′–PLGA(FITC), Lane3: Fab′–PLGA(FITC) treated with DTT, Lane4: PLGA(FITC).

To exam the reducible cleavability of a disulfide bond between siRNA and PLGA, siRNA**–**PLGA conjugate was treated with a reducing agent, DTT, and the cleaved siRNA was visualized by 4% agarose gel electrophoresis and ethidium bromide staining (Fig. 2a, lane 3). Furthermore, the siRNA**–**PLGA micelles showed retarded migration in the gel (Fig. 2a, lane 2) as compared to naked siRNA (Fig. 2a, lane 1). These results suggest that intracellularly delivered siRNA–PLGA conjugate micelles could be disassemble in the cytoplasm with subsequently liberating free siRNA for RNAi mechanism. While, to exam the exist of Fab′ in Fab′**–**PLGA and the reducible cleavability of a disulfide bond between Fab′ and PLGA, Fab′**–**PLGA conjugate was treated with a reducing agent, DTT, and Fab′ in Fab′**–**PLGA and the cleaved Fab′ was visualized by SDS-PAGE and Ag staining (Fig. 2b, lane 2 and 3, respectively). The light chain and Fab′ without the light chain were visualized at 25 kDa as the cleaved Fab′ in lane 3. However, retarded migration in the gel of Fab′–PLGA (lane 2) compared to naked Fab′ (lane 1) did not be observed such as siRNA–PLGA. Therefore, SDS-PAGE was performed to exam the reducible cleavability of a disulfide bond between Fab′ and PLGA, free FITC labeling PLGA was visualized by FITC labeling PLGA (Fig. 2c, lane 3 and 4). It was clarified that the fluorescence intensity was attenuated when micelles were formed (Fig. 2b, lane 2). The fluorescence intensity of Fab′**–**PLGA micelles was also attenuated (Supplementary Fig. S2b). While, the fluorescence intensity derived free FITC labeling PLGA was detected in F(ab′)2 and PLGA mixture group without covalent bond (Supplementary Fig. S2b, lane 1). These results supported to exist of Fab′**–**PLGA conjugate via covalent bond.

### Confirmation of Fab′ binding activity against its antigen using flow cytometry

F(abʹ)2 was detected by western blotting after sodium dodecyl sulphate–polyacrylamide gel electrophoresis (SDS-PAGE) was performed using a 150-kDa band and was purified using a commercially available F(abʹ)2 preparation kit (Supplementary Fig. S1a,b). A functional binding analysis was performed using flow cytometry (Supplementary Fig. S1c). The Fabʹ that was used as a component of the PLGA hybrid retained binding activity against human CD71 expressed on the surface of CEMx174 cells and was similar to fully expressed IgG activity levels.

### Evaluation of siRNA–PLGA/Fabʹ–PLGA mixing ratios based on intercellular uptake efficiency

The siRNA–PLGA and Fabʹ–PLGA mixing ratios were evaluated using flow cytometry based on the siRNA intracellular uptake efficiency. The fluorescence intensity of cells that were treated with various Fabʹ–PLGA ratios to prepare a sample that was entirely (Alexa488-siRNA)-PLGA was measured after 24 h of incubation. Microscopic photographs were taken before the fluorescence was measured using flow cytometry.

**Figure 3:**
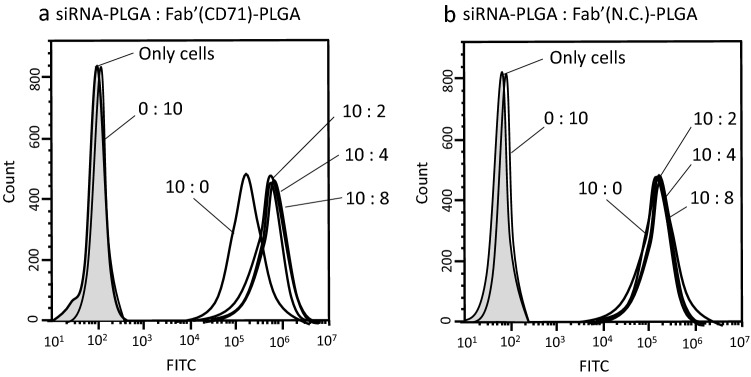
Flow cytometric measurement of the fluorescence intensity of CEMx174 cells that were transfected Alexa488-siRNA using various mixing weight ratios of siRNA–PLGA and Fabʹ–PLGA mixed micelles. The mixing volume ratio at the side of peak in the figure is shown as (Alexa488-siRNA)–PLGA:Fabʹ(anti CD71 Ab)–PLGA (**a**) and (Alexa488-siRNA)–PLGA:Fabʹ(normal mouse IgG as a negative control)–PLGA (**b**), respectively. The figures were created using NovoCyte Software NovoExpress version. 1.2.4.

Figure 3a shows that the peak for siRNA–PLGA/Fabʹ–PLGA mixed micelles shifted more to the right compared with the peak for Fabʹ–PLGA micelles. However, there were no significant differences in intracellular siRNA uptake efficiency between the various mixing ratios of the Fabʹ–PLGA and siRNA–PLGA; this was attributed to the reactivity of Fabʹ to CD71 highly expressed on the CEMx174 cell surface protein. Based on these results and those of a previous study using a ratio of 25% antibody fragment to micelles mol%^18^, the Fabʹ–PLGA-to-siRNA–PLGA mixing ratio of 25% was used in this study as the minimum concentration for sufficient functionality. In the case of siRNA/Fabʹ–PLGA mixed micelles using the control IgG (non-specific activity to CD71; N.C.), the peak was overlapped by the peak of siRNA–PLGA (10:0) (Fig. 3b). These results prove that the use of CD71-specific Fabʹ used in this preparation improves the specific intracellular uptake of target cells.

### Size and surface charge of siRNA–PLGA micelles and siRNA–PLGA/Fabʹ–PLGA mixed micelles

The hydrodynamic sizes and surface charges of siRNA–PLGA micelles and siRNA–PLGA/Fabʹ–PLGA mixed micelles were analyzed using a dynamic light-scattering technique. As shown in Table 1, the mean diameter and zeta potential of siRNA–PLGA micelles were 113.3 ± 0.09 nm and − 22.1 mV, respectively, and the mean diameter and zeta potential of siRNA–PLGA/Fabʹ–PLGA mixed micelles were 112.8 ± 0.30 nm and − 42.5 mV, respectively. The size distributions of each conjugate micelles were observed sharply in each conjugate micelle’s profiles (Supplementary Fig. S3). These data were supported that each conjugate formed micelle structures. Fabʹ–PLGA micelles were also prepared as a control. The mean diameter and zeta potential of Fabʹ–PLGA micelles were 170.9 ± 0.26 nm and − 68.5 mV, respectively.

**Table 1:** Particle sizes and zeta potential values of siRNA–PLGA micelles, siRNA–PLGA/Fabʹ–PLGA mixed micelles, and Fabʹ–PLGA micelles. Data are presented as the mean ± SD (n = 3).

Sample	Particle size (nm)	Zeta potential (mV)
siRNA–PLGA	113.3 ± 0.09	− 22.1
siRNA–PLGA/Fab′–PLGA	112.8 ± 0.30	− 42.5
Fab′–PLGA	170.9 ± 0.26	− 68.5

### Cyclin B1 expression levels in CEMx174 cells treated with siRNA–PLGA micelles and siRNA–PLGA/Fabʹ–PLGA mixed micelles

**Figure 4:**
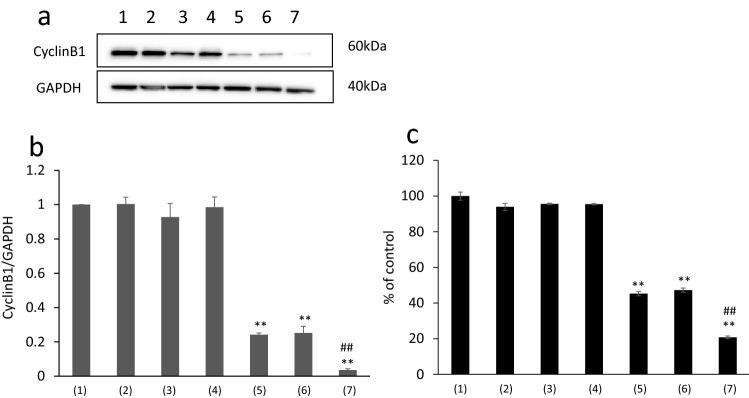
(**a**) Blotting images of Cyclin B1 levels in CEMx174 cells treated with siRNA–PLGA micelles and siRNA–PLGA/Fabʹ–PLGA mixed micelles and (**b**) its western blotting analysis. (1) control, (2) siRNA (N.C.), (3) siRNA, (4) siRNA (N.C.)–PLGA, (5) siRNA–PLGA, (6) siRNA–PLGA/Fabʹ (N.C.)–PLGA, (7) siRNA–PLGA/Fabʹ–PLGA. Data are presented as the mean ± SD (n = 5). **p < 0.01 versus control. ^#*#*^*p* < 0.01 versus siRNA–PLGA (Bonferroni test/ANOVA). (**c**) Cell viability of CEMx174 cells treated with siRNA–PLGA micelles and siRNA–PLGA/Fabʹ–PLGA mixed micelles. (1) control, (2) siRNA (N.C.), (3) siRNA, (4) siRNA (N.C.)–PLGA, (5) siRNA–PLGA, (6) siRNA–PLGA/Fabʹ(N.C.)–PLGA, (7) siRNA–PLGA/Fabʹ–PLGA. Data are presented as the mean ± SD (n = 3). **p < 0.01 versus control. ^##^*p* < 0.01 versus siRNA–PLGA group (Bonferroni test/ANOVA).

siRNA sequences that exhibited potent knockdown effects in a dose-dependent manner when delivered with transfection reagents were selected for subsequent experiments using micellar delivery without transfection agents. The cyclin B1 levels in CEMx174 cells treated with siRNA–PLGA micelles and siRNA–PLGA/Fabʹ–PLGA mixed micelles were then evaluated with the help of western blotting. siRNA–PLGA micelles significantly suppressed cyclin B1 expression compared with the control, as did siRNA–PLGA/Fabʹ–PLGA mixed micelles (Fig. 4a,b). Moreover, siRNA (negative control; N.C.)–PLGA micelles and Fabʹ–PLGA micelles, which do not include cyclin B1-targeting siRNA, did not suppress cyclin B1 expression as seen in the control group. Especially, siRNA–PLGA/Fabʹ–PLGA mixed micelles were significantly suppressed cyclin B1 expression compared with siRNA–PLGA/Fabʹ(N.C.)–PLGA mixed micelles. These results suggested that mixed micelle’s targeting effect was caused by CD71-specific Fab′ activity.

In the evaluation of the cyclin B1 knockdown effect using the CEMx174 cell line, mixed micelles showed a significant protein knockdown effect compared with non-mixed micelles.

### Cell viability assay

Cell viability was evaluated to determine the effects of cyclin B1 downregulation on cell proliferation. Treatment with siRNA–PLGA micelles and siRNA–PLGA/Fabʹ–PLGA mixed micelles, particularly the latter, significantly suppressed cell proliferation compared with the control (Fig. 4c). These results were correlated with data of suppression of cyclin B1 expression in CEMx174 cells treated with mixed micelles (Fig. 4).

### Cell accumulation of the siRNA–PLGA hybrid or Fabʹ–PLGA hybrid using labeled PLGA in the CEMx174 cell line culture medium

**Figure 5:**
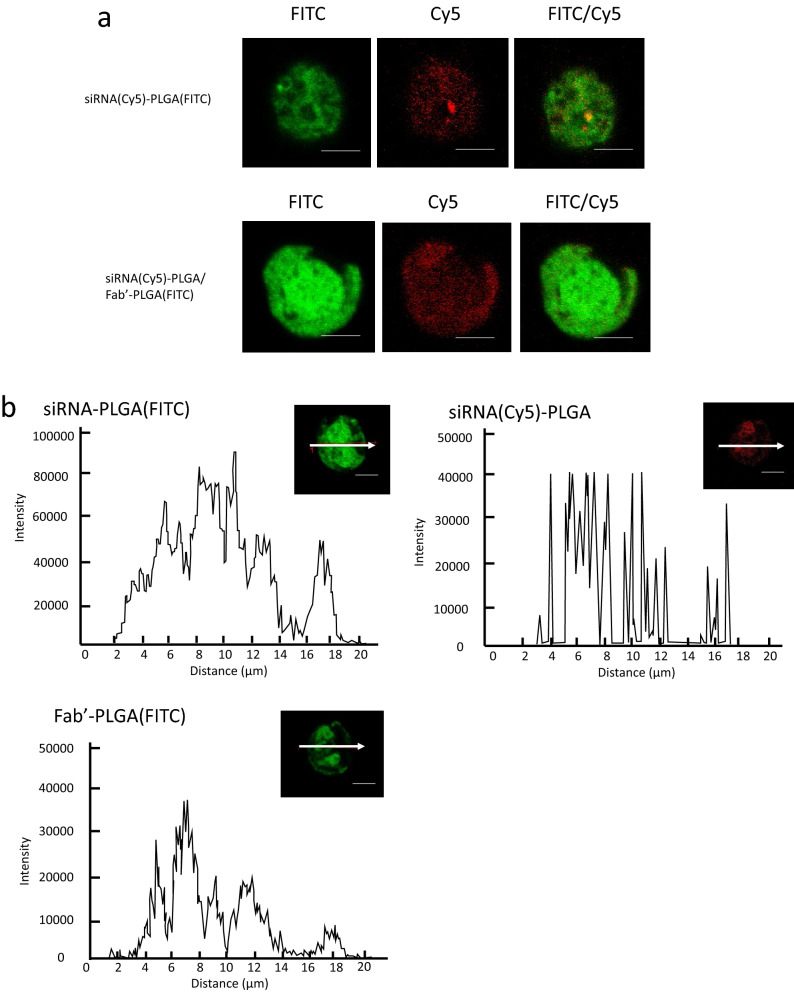
(**a**) Intracellular uptake of siRNA–PLGA, as shown in the upper line labeled with Cy5 or FITC, and siRNA–PLGA/Fabʹ–PLGA mixed micelles consisting of (Cy5-labeled siRNA)-PLGA and Fabʹ-(FITC-labeled PLGA), as shown in lower line in CEMx174 cells using a confocal laser microscope. (**b**) Image analysis of the fluorescence intensity of CEMx174 cells in the cross section with a white arrow using three types of labeling conjugates to evaluate the localization of these conjugates. The scale bar is 10 µm. These images were captured by LSM 710 instrument and figures of image analysis were created using LSM Software ZEN 2009.

It was considered that Fabʹ–PLGA could be present only on the cell surface due to antibody–antigen reaction. From the results of protein quantification using western blotting (Fig. 4a), we can see that siRNA was delivered efficiently to the cells via the mixed micelle system. However, the localization of these hybrids cannot be determined using flow cytometry alone (Fig. 3). Further, to evaluate the cell accumulation of the micelles when added to the cells, fluorescence intensity was measured using fluorescently labeled micelles comprising fluorescein isothiocyanate (FITC)-labeled PLGA via a confocal laser scanning microscope. Localization was observed by a confocal microscope to evaluate the differences between mixed and non-mixed micelles conjugating in the four types of PLGA (Fig. 5a,b, respectively). The fluorescence intensity induced by siRNA or PLGA in the two types of micelles was detected not only on the cell surface but also inside the cells.

## Discussion

From our previous study^6^ and results of other research groups^19, 20^, we considered that “micelle structure” was important to deliver siRNA into the cell as an effective carrier but not “monomer conjugate”. Therefore, it was necessary to synthesize conjugates for the purpose of micelle formulation. From these reasons, hydrophilic siRNA and Fab′ needed to be covalently linked to hydrophobic PLGA, respectively. In addition, this mixed micelle system can change the mixing ratio within a certain range compared with a single polymer molecule whose mixing ratio of siRNA and Fab′ cannot be changed. This point has an advantage in the mixed micelles system when it is assumed to be put into practical use.

In this study, we confirmed that the antibodies did not lose their activity due to fragmentation as shown in Supplementary Fig. 1c, although fragmentation might inhibit their activity depending on the method and antibody stability^21^. Thus, the mixed micelle formulation prepared in this study is designed as a novel dosage form that recognizes target molecules like CD71 wherein the antibody activity is maintained.

The average particle size of the mixed micelles prepared in this study was approximately 110 nm, and the surface charge was approximately − 30 mV, but the Fabʹ–PLGA micelle particle size was larger than other micelles. The particle size difference was suggested to result from the intermolecular force of the hybrid during micelle formation; this force varies greatly depending on the type of monomer and solvent^22^. The mixed micelle is almost the same size as a single non-mixed micelle and mixing does not interfere with the cell permeation efficiency.

From the results shown in Fig. 4 or those of our previous study^6^, approximately 100 nm particles, including siRNA–PLGA micelles, undergo endocytosis. Endocytosis is thought to be promoted by attracting more micelles to cells, which occurred due to the binding force of Fabʹ with CD71. Thus, the siRNA transfection efficiency of the cells increased. It was speculated that not only endocytosis but also the promoting of CD71 internalization enhances the knockdown effect as an additive action^23, 24^. Therefore, it was suggested that the intracellular uptake of mixed micelles involves target receptor internalization, which then has an additive effect on endocytosis promotion by the mixed micelle with an antibody fragment against CD71.

Cyclin B1, a target protein of siRNA, was significantly involved in the cell cycle and regulated cell proliferation^25^. Cyclin B1 may also be a key target for anti-proliferative strategies, and siRNA used in cyclin B1 targeting approaches has been proposed as a useful antiproliferative therapy^26–28^. Therefore, these results have a positive correlation between the protein expression level and cell proliferation and are appropriate. Furthermore, it is assumed that protein knockdown by siRNA leads to the suppression of cell growth.

These results show that micelles consisting of siRNA-loaded PLGA conjugates were taken up into the cell by endocytosis, and siRNA was reliably delivered intracellularly. Furthermore, the Fabʹ–PLGA conjugate was also incorporated into the cell as a micelle particle rather than staying only on the cell surface. From these results, it was determined that the mixed micelles recognized target cells and efficiently delivered siRNA into the cells without inhibiting the intercellular uptake of micelle particles. It has been suggested that mixed micelles be used as novel drug carriers that recognize target cells or as an efficient drug-delivery device to deliver siRNA into the cells.

To our knowledge, this was the first study to show that siRNA–PLGA/Fabʹ–PLGA mixed micelles can effectively deliver siRNA to cancer cells and recognize cancer cells in vitro. In addition, our strategy for targeting cancer cells using antibody-based drugs could offer new information about developing nucleic acid medications that use siRNA as a therapeutic approach without the side effects associated with anticancer drugs. This study targeted CEMx174, a lymphocyte. Therefore, the mixed micelle proposed in this study is expected to target solid tumors or floating cells like blood cancers or immune cells by customizing the antibody target. The design formulated for targeting in this study can be applied to various diseases because the amino acid sequence of Fabʹ and the base sequence of siRNA for targeting can be customized according to the therapeutic approach required for a particular disease. In this study, we focused on evaluation in vitro to prove CD71-specific Fab′ activity of mixed micelle systems efficiently taking advantage of the high affinity between CEMx174 cells and anti-CD71 antibody. However, it is an important task to clarify its usefulness in vivo in order to further emphasize the Fab′ targeting role in mixed micelle systems and establish a basis for its practical application in the future. That is why, we are planning further experiments to prove in vivo therapeutic effects of this mixed micelle systems using mice model in the future study based on our in vitro data.

## Methods

### Materials

Poly(d,l-lactic-co-glycolic acid) (PLGA7510; M_W_, 10,000) was purchased from Wako Pure Chemical Industries, Ltd. (Osaka, Japan). Human cyclin B1-specific siRNA, which was modified with a thiol group at the 3ʹ end of the sense or antisense strand, was purchased from Thermo Fisher Scientific, Inc. (Waltham, MA, USA). The siRNA sequences were as follows: cyclin B1 siRNA sense: 5ʹ-GGC GAA GAU CAA CAU GGC ATT-3ʹ; and cyclin B1 siRNA antisense: 5ʹ-UGC CAU GUU GAU CUU CGC CTT-3ʹ^25^. Negative control siRNA was also purchased from Thermo Fisher Scientific, Inc. (Waltham). Fetal calf serum (FCS) and Roswell Park Memorial Institute (RPMI) 1640 were purchased from Gibco (Cergy-Pontoise, France). Anti-human CD71 monoclonal antibody, phycoerythrin (PE) anti-human CD71 antibody (Clone: OKT9) and were purchased from Thermo Fisher Scientific Inc. Normal mice IgG1 kappa monoclonal, which was used as the control IgG, was purchased from Abcam (ab91353, Cambridge, UK). A Pierce IgG F(abʹ)2 preparation kit was purchased from Thermo Fisher Scientific Inc. Cell Counting Kit-8 (CCK-8) reagent was purchased from Dojindo Laboratories (Tokyo, Japan). Alexa488-labeled siRNA was purchased from Thermo Fisher Scientific, Inc. (Waltham). Cy5-labeled siRNA was purchased from Nippon Gene (Toyama, Japan). FITC-labeled PLGA was synthesized by the NARD Institute, Ltd. (Amagasaki, Japan).

### Preparation of siRNA–PLGA micelles

The siRNA–PLGA hybrid block copolymer was synthesized as previously described^6^. Antisense and sense 3ʹ thiol-modified siRNAs were conjugated to PLGA-3-(2-pyridyldithio) propionyl hydrazide (PDPH) via disulfide exchange reaction. The synthesized siRNA–PLGA hybrids were expected to form self-assembled micelles in aqueous solutions, thereby resulting in a substantially increased charge density of clustered siRNAs on the outer shell (Fig. 1a,c).

### Preparation and isolation of the F(abʹ)2 fragment from the whole antibody

A F(ab)2 preparation kit was used to cleave F(abʹ)2 from the whole antibody in accordance with the manufacturer’s protocol. In this study, anti-human CD71 antibody was used as a model for targeting the Fabʹ fragment because CD71 is expressed highly in the CEMx174 cell line. The yield solution was prepared in SDS-PAGE loading dyes and analyzed using SDS-PAGE.

### Preparation of Fabʹ–PLGA micelles and siRNA–PLGA/Fabʹ–PLGA mixed micelles

F(abʹ)2 fragments of the antibody in phosphate-buffered saline (PBS; 0.5 mg/mL, 400 µL) were mixed with dithiothreitol (DTT) (0.5 mM, 240 µL) and incubated at 37 °C for 30 min^12^ to reduce F(abʹ)2 fragments into Fabʹ fragments possessing a thiol group. The Fabʹ fragment solution was then conjugated to PLGA–PDPH via disulfide exchange reaction in the same manner that was used to prepare the siRNA–PLGA hybrid. The siRNA–PLGA and Fabʹ–PLGA hybrid solutions were mixed at a volume ratio of 4:1 in accordance with previous methods^19^ (Fig. 1b,c).

### Characterization of siRNA–PLGA conjugate and Fab′–PLGA conjugate

The resultant siRNA**–**PLGA conjugates was separated by 4% agarose gel (NuSieve GTG agarose, Lonza Bioscience, Bazel, Switzerland) electrophoresis (100 V, 40 min) and ethidium bromide. To confirm the cleavage of reductive disulfide bond in siRNA–PLGA conjugate, 10 mM of DTT was treated 14.5 pmol (as content of siRNA) of siRNA–PLGA conjugate in PBS solution (pH 7.4) for 15 min at room temperature and cleaved siRNA was analyzed by gel electrophoresis. Fab′–PLGA conjugate using FITC labeling PLGA was separated by SDS-PAGE. To confirm the cleavage of reductive disulfide bond in Fab′–PLGA conjugate, 10 mM of DTT was treated 0.2 µg (as content of Fab′) of Fab′–PLGA conjugate in PBS solution (pH 7.4) for 15 min at room temperature. After SDS-PAGE, Fab′ in Fab′–PLGA and FITC derived FITC labeling PLGA were detected by Ag staining and transilluminator (Lti-ExLB LED 485 nm, BioTools Inc., Takasaki, Japan), respectively, using same gel. Images of gel electrophoresis stained by ethidium bromide and Ag were captured using an Alpha Innotech Fluorchem 8900 Imager.

### Morphology and surface charge of the siRNA–PLGA micelles and siRNA–PLGA/Fabʹ–PLGA mixed micelles

The sizes and surface charges of the siRNA–PLGA micelles and siRNA–PLGA/Fabʹ–PLGA mixed micelles were measured using a dynamic light-scattering instrument (Zetasizer; Malvern Instruments, Ltd., Malvern, UK). Both these types of micelles (300 pmol) were dissolved in 500 μL distilled water. The effective hydrodynamic diameters and zeta potentials of these micelles were measured in triplicate.

### Cell culture

The human T–B hybrid cell line CEMx174 was a gift from the American Type Culture Collection (ATCC; catalog CRL-1991, Rockville, Maryland, USA). CEMx174 cells were cultured in an RPMI 1640 medium (Gibco) supplemented with 10% FCS at 37 °C in an atmosphere of 95% air and 5% CO_2_.

### Cyclin B1 gene silencing by siRNA–PLGA micelles or siRNA–PLGA/Fabʹ–PLGA mixed micelles without a transfection reagent

CEMx174 cells were seeded in a 24-well plate at a density of 5 × 10^5^ cells per well to measure the relative cyclin B1 expression by western blotting. The siRNA–PLGA micelles or siRNA–PLGA/Fabʹ–PLGA mixed micelles (80 pmol each based on the siRNA content) were added to the well. After 48 h of incubation, the cells were lysed with 1% (w/v) Triton X-100 solution in PBS and centrifuged to remove cell debris.

### Western blot analysis

To detect the cyclin B1 protein, cells were lysed in a lysis buffer. The total protein concentrations of the lysates were measured using bicinchoninic acid (BCA) assay (Pierce Biotechnology, Rockford, IL, USA). Cell lysates containing equal amounts of protein were separated by SDS-PAGE (Bio-Rad, Hercules, CA, USA), and protein bands were transferred to polyvinylidene difluoride membranes (Bio-Rad). The membranes were blocked with Blocking One (Nacalai Tesque, Inc., Kyoto, Japan) overnight at 4 °C and then incubated for 1 h at 22–25 °C with anti-cyclin B1 antibody (ab2949; Abcam, Cambridge, UK) or an anti-glyceraldehyde 3-phosphate dehydrogenase antibody (ACR001PT; Acris Antibodies, Inc., San Diego, CA, USA) at a 1:1000 and 1:10,000 dilution, respectively, in a blocking solution. After washing three times, the membranes were incubated for 1 h at 22–25 °C with a secondary antibody (horseradish peroxidase-conjugated species-specific antibody). Immunoreactive bands were visualized with ImmunoStar LD (Wako Pure Chemical Industries). Western blotting chemiluminescence was quantified using an Alpha Innotech Fluorchem 8900 Imager and AlphaEase Software version 3.2.3.

### Cell viability assay

CyclinB1 gene knockdown effect on the proliferation of CEMx174 cells was evaluated using the CCK-8 reagent. Briefly, 1 × 10^4^ cells in 100 µL RPMI 1640 containing 10% FCS were seeded into 96-well plates. Each well was then treated with 50 µL of micelles for 48 h or with 50 µL PBS as a control. Then, 15 µL CCK-8 reagent was added to each well, and the cells were further incubated at 37 °C for 2 h. Absorbance was measured using a microplate reader at test and reference wavelengths of 450 and 655 nm, respectively, to evaluate the relative viability of the treated cells.

### Flow cytometry

#### Evaluation of the binding activity of the antibody fragment

IgG, F(abʹ)2, or Fabʹ was added to CEMx174 cells blocked by 40% normal goat serum at a density of 5 × 10^5^ cells per tube. After a 30-min incubation, cells were washed using a washing buffer, and PE-labeled secondary antibody against IgG was added to the cells. After a 30-min incubation, the fluorescence intensity of the cells was determined using a flow cytometer (NovoCyte, ACEA Biosciences, Inc., San Diego, CA, USA).

#### Evaluation of the mixing volume ratio in siRNA–PLGA/Fabʹ–PLGA mixed micelles

CEMx174 cells were seeded in a 24-well plate at a density of 5 × 10^5^ cells per well. siRNA–PLGA mixed with Fabʹ–PLGA was added to the well at a volume ratio of 0:0, 10:0, 10:2, 10:5, 10:8, or 0:10. The 10-volume ratio of siRNA–PLGA was determined to be 80 pmol based on the siRNA content, whereas the 8-volume ratio of Fabʹ–PLGA was 5 µg based on the Fabʹ content. Cells were used to evaluate the fluorescence intensity after 24 h of incubation. The fluorescence intensity of Alexa488-siRNA was detected using a flow cytometer.

### Localization of the siRNA–PLGA or Fabʹ–PLGA conjugate consisting of mixed micelles after cellular uptake into the CEMx174 cells

CEMx174 cells were seeded in a 24-well plate at a density of 5 × 10^5^ cells per well. The four types of siRNA–PLGA/Fabʹ–PLGA mixed micelles were added to the well to evaluate whether each part of the micelle was taken up into the cells. After 24 h of incubation, the cells were used to evaluate the fluorescence intensity. In this evaluation, Cy5-labeled siRNA or FITC-labeled PLGA was used to prepare three types of a siRNA–PLGA hybrids, (Cy5-labeled siRNA)-PLGA, siRNA-(FITC-labeled PLGA), and Fabʹ-(FITC-labeled PLGA). The fluorescence intensity of Cy5 and FITC was observed using a confocal microscope (LMS710, Carl Zeiss, Oberkochen, Germany) with a 40 × objective lens to evaluate the localization of the fluorescence intensity in the cells by slicing to distinguish between the surface and cell interior. Image analysis was performed using LSM Software ZEN 2009 (Carl Zeiss).

### Statistical analysis

Values were expressed as mean ± standard deviation (SD) (n = 3–5). To determine the differences among the groups, data were evaluated for statistical significance using the Bonferroni test. Overall significance was determined using a one-way repeated measures analysis of variance (ANOVA). A *p*-value of < 0.05 was considered to be statistically significant.

## Supplementary Information

Supplementary Figures.

## Data Availability

The datasets generated and/or analyzed during the current study are available from the corresponding author on reasonable request.
