# Supplementary material for: Preparation of siRNA–PLGA/Fabʹ–PLGA mixed micellar system with target cell-specific recognition
Source: Sci Rep. 2021 Aug 18;11:16789. doi: 10.1038/s41598-021-96245-3 (PMC8373956; doi:10.1038/s41598-021-96245-3)
Supplement: Supplementary file 1 — Supplementary Figures. [file 41598_2021_96245_MOESM1_ESM.pdf]

**Supplementary Information**

# Preparation of siRNA–PLGA/Fab′–PLGA mixed micellar system with target cell-specific recognition

Mai Hazekawa[1*], Takuya Nishinakagawa[1], Takeshi Mori[2], Miyako Yoshida[2], Takahiro Uchida[2] and Daisuke Ishibashi[1]

[1]Department of Immunological and Molecular Pharmacology, Faculty of Pharmaceutical Sciences, Fukuoka University, Fukuoka, Japan

[2]Department of Clinical Pharmaceutics, Faculty of Pharmaceutical Sciences, Mukogawa Women's University, Hyogo, Japan

**Supplementary Figure S1**

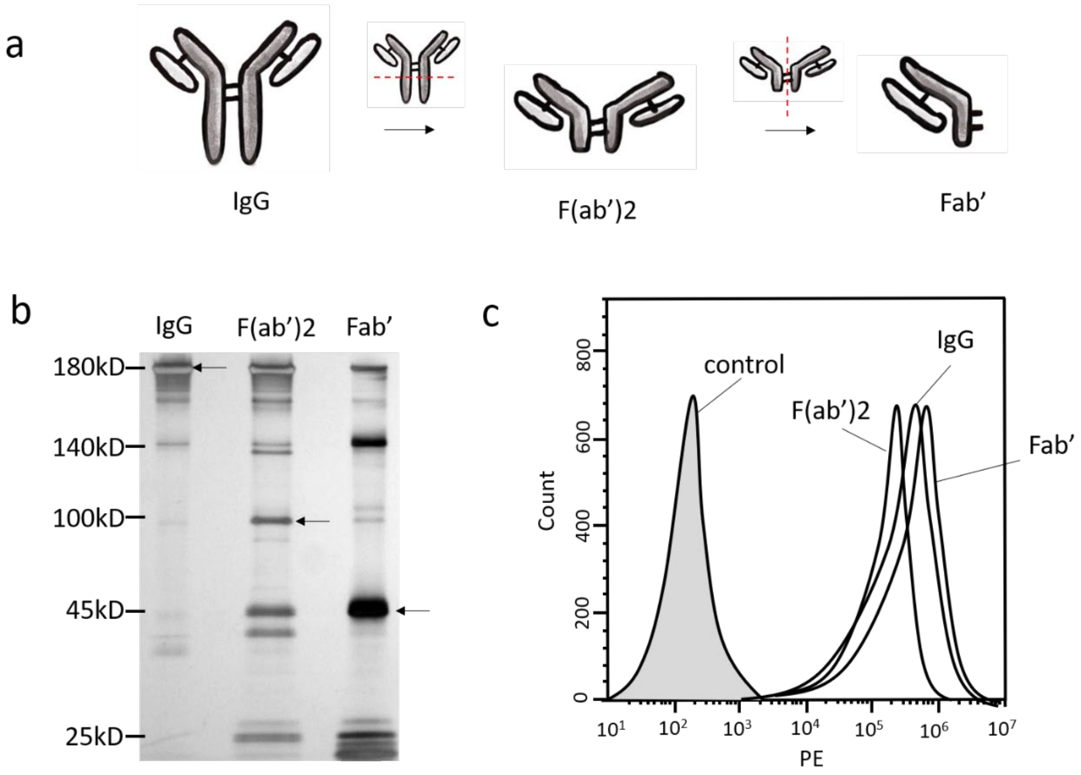


**F(ab')2 and Fab' structure obtained by fragmentation of IgG and existence confirmation and their activity evaluation for the antigen**

(a) Shape of the fragmentation of IgG; (b) Detection of IgG fragmentation using SDS-PAGE; (c) Evaluation of the binding activity of antibody fragments used in this study using a flow cytometer.

**Supplementary Figure S2**

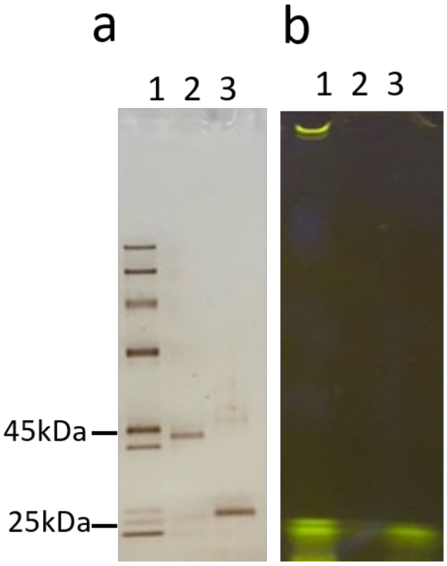


**Characterization of siRNA-PLGA and Fab'-PLGA conjugate via covalent bond** (a) Fab' visualized by Ag staining in the gel after SDS-PAGE. Lane1: F(ab')2 and PLGA(FITC) mixture, Lane2: Fab'–PLGA(FITC), Lane3: Fab'–PLGA(FITC) treated with DTT (b) FITC visualized by transilluminator in the gel after SDS-PAGE. Lane1: F(ab')2 and PLGA(FITC) mixture, Lane2: Fab'–PLGA(FITC), Lane3: Fab'–PLGA(FITC) treated with DTT.

**Supplementary Figure S3**

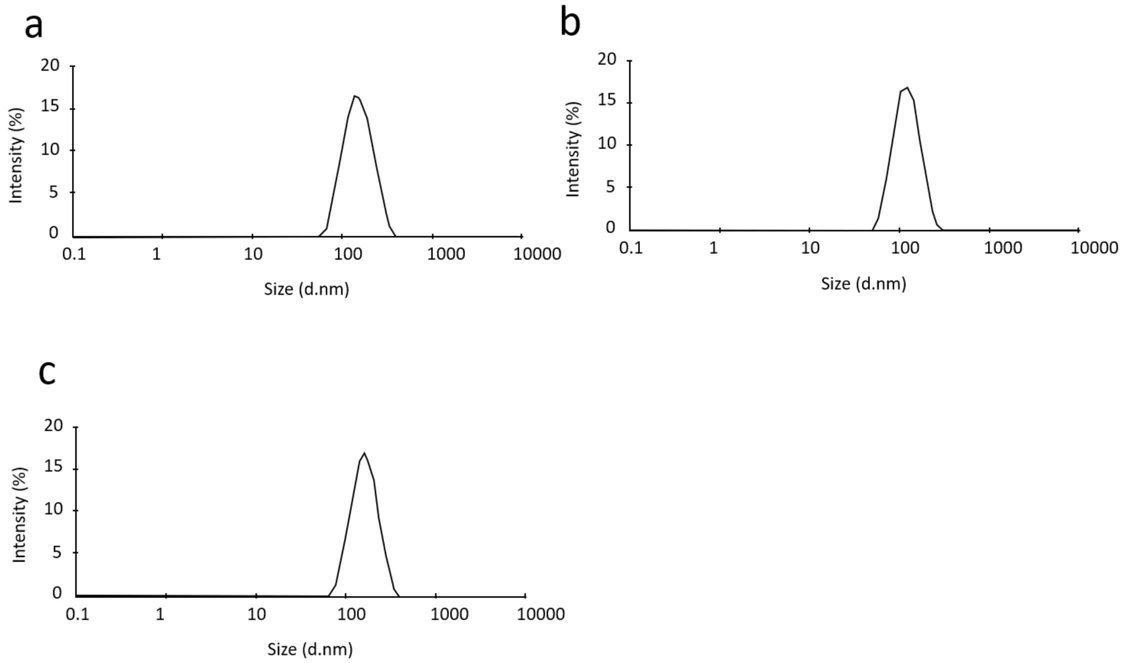


**The size distribution of each conjugate micelles.** (a) siRNA–PLGA conjugate micelles (b) siRNA–PLGA/ Fab'–PLGA mixed micelles (c) Fab'–PLGA conjugate micelles.

**Supplementary Figure S4**

S1b

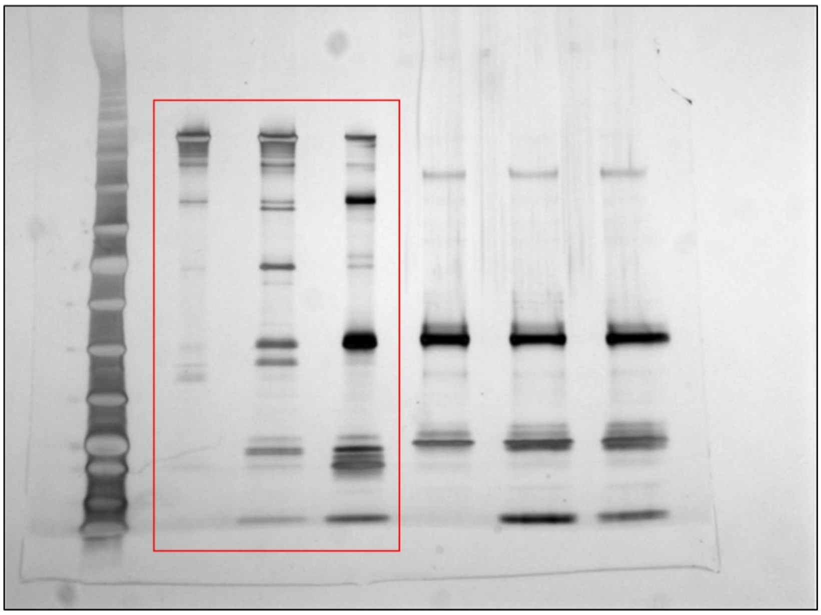

S2a, Ag stein of SDS-PAGE

S2b, Fluorescence visualized by transilluminator of SDS-PAGE

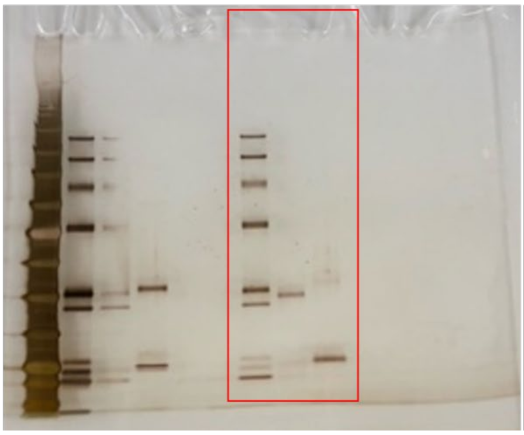

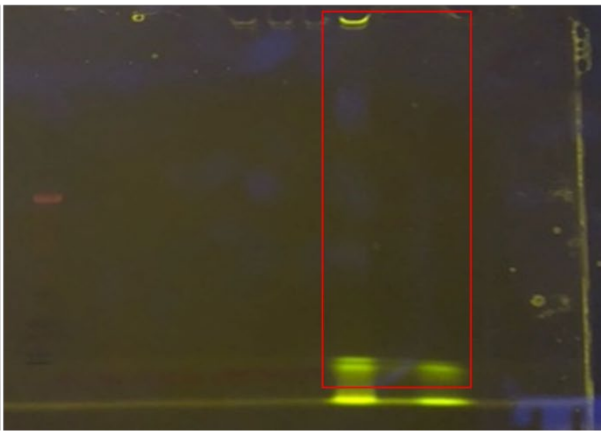

Original uncropped images of immunoblotting of main figures.

**Supplementary Figure S4 (continued)**

Fig. 2a

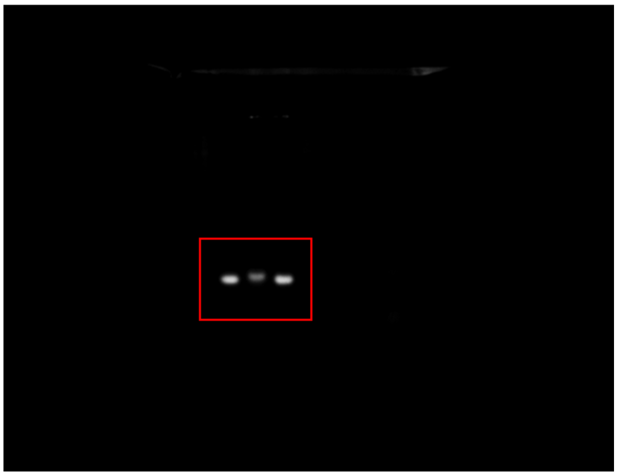

Fig. 2b

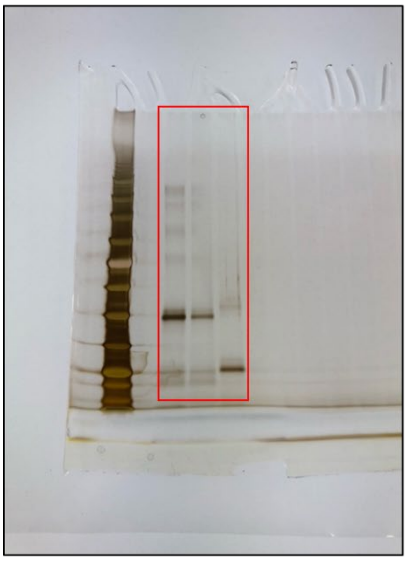

Fig. 2c

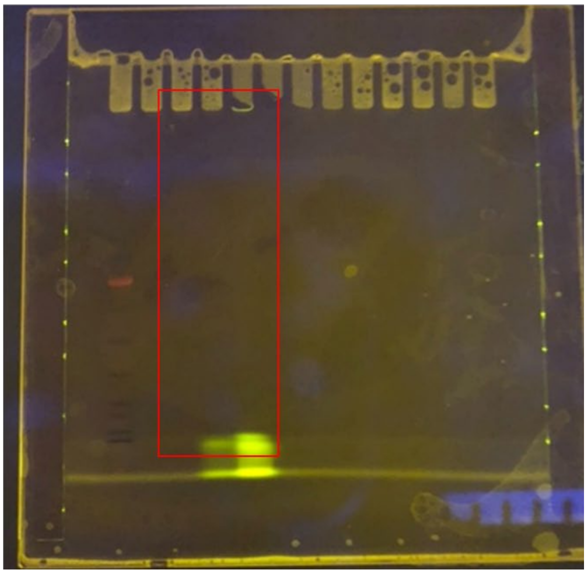

Original uncropped images of immunoblotting of main figures.

**Supplementary Figure S4 (continued)**

Fig. 4a, CyclinB1

Exposure time 60 sec

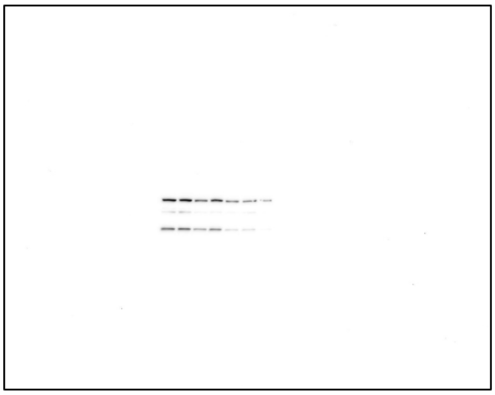

Exposure time 120 sec

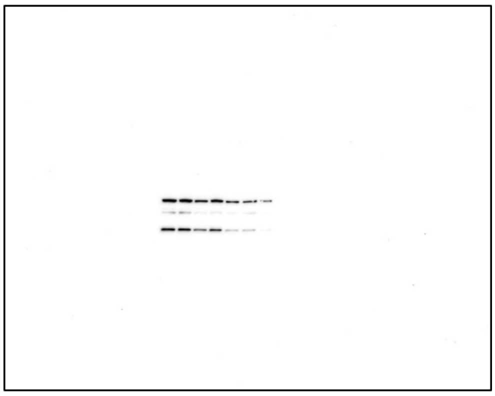

Exposure time 180 sec

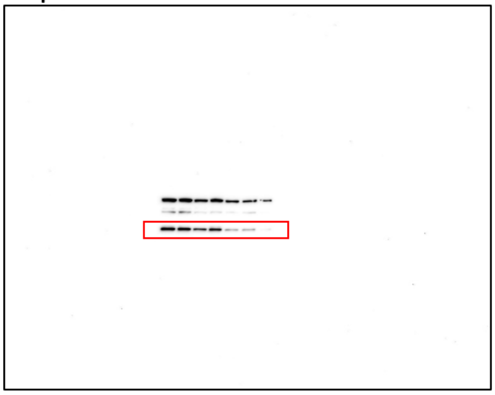

Original uncropped images of immunoblotting of main figures.

**Supplementary Figure S4 (continued)**

Fig. 4b, GAPDH

Exposure time 10 sec

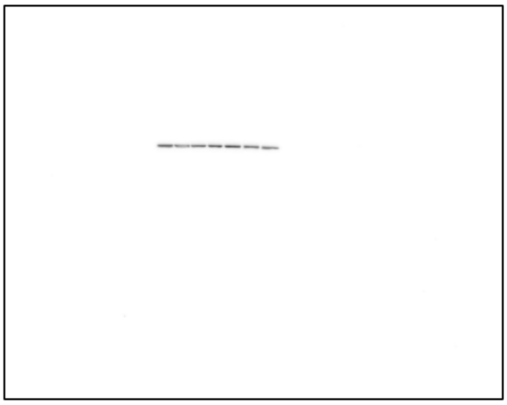

Exposure time 20 sec

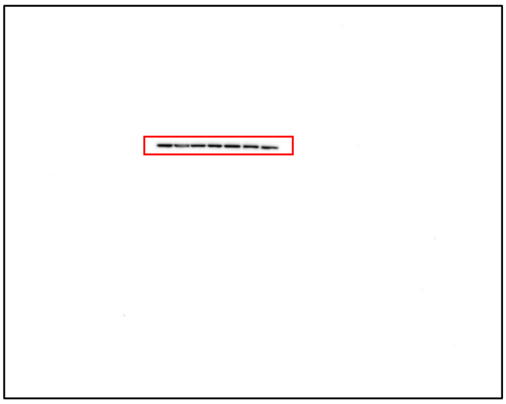

Exposure time 30 sec

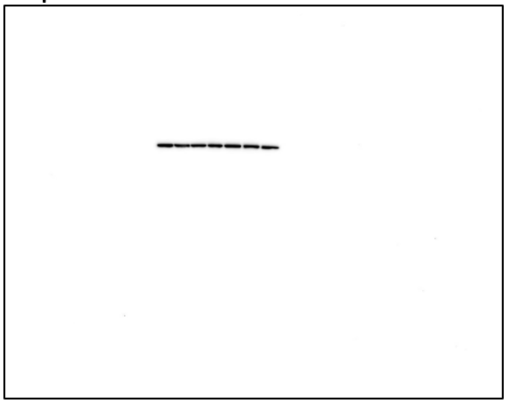

Original uncropped images of immunoblotting of main figures.
